# Macrophage activation syndrome in a Sudanese child: a case report from Sudan

**DOI:** 10.1097/MS9.0000000000001900

**Published:** 2024-03-06

**Authors:** Maha Abdalla Yahia Elhassan, Roua Mohammedali Hussain Idres, Baraa Mohamed Salaheldin Mohamed Elhassan, Naema Siddiq Banaga Siddiq, Rayan Abdelwahab Osman Mohammed, Mohammed Mahmmoud Fadelallah Eljack, Ghassan E. Mustafa, Lina Hemmeda, Khabab Abbasher Hussien Mohamed Ahmed

**Affiliations:** a Faculty of Medicine, University of Khartoum; b Faculty of Medicine, University of AlNeelain, Khartoum; c Faculty of Medicine and Health Sciences, University of Bakht Alruda, Ad Duwaym, Sudan

**Keywords:** macrophage activation syndrome, idiopathic juvenile arthritis, paediatric, rheumatology, Sudan

## Abstract

**Introduction and importance:**

Macrophage activation syndrome (MAS) is a severe form of hemophagocytic lymphohistiocytosis that is frequently associated with either a flare-up of rheumatologic diseases, or infection and is characterized by intermittent fever, organomegaly, and multisystem dysfunction. Early diagnosis and treatment are crucial for outcome improvement.

**Case presentation:**

The authors present a 9-year-old male with systemic onset juvenile idiopathic arthritis who presented with fever, vomiting, and nose bleeding, as well as being jaundiced, and having hepatomegaly and ascites. Pancytopenia, hepatic dysfunction, and elevated ferritin levels were discovered, along with negative virological and immunological tests. He was given broad-spectrum antibiotics, and a high-dose steroid showed a good response, and he was discharged about a week later.

**Clinical discussion:**

It is hypothesized that decreased natural killer cells’ function could lead to the inability to clear the infection, and subsequent lymphocytes-induced macrophages activation. Despite being beneficial in this case, steroids led to no improvement in other similar cases.

**Conclusion:**

MAS is a real life-threatening complication for patients with systemic Juvenile idiopathic arthritis (sJIA), and early diagnosis and prompt initial treatment can both offer a favourable result against such syndrome.

## Introduction

Highlights

- Macrophage activation syndrome (MAS) is a real life-threatening complication for patients with systemic Juvenile idiopathic arthritis.
- Intermittent fever, organomegaly, and multisystem dysfunction are the hallmark findings for suspecting MAS.
- High-dose steroid showed a good response in management of MAS.
- Early diagnosis and prompt initial treatment can both offer a favourable result against such syndrome.

Hemophagocytic lymphocytosis (HLH) is regarded as a rare but fatal disorder with extremely high mortality rates. It is often the result of genetic defects in immune system function or due to infectious diseases (such as Epstein-Barr virus, Cytomegalovirus, Parvovirus B19), tumours, and autoimmune disorders^1^.

Macrophage activation syndrome (MAS), a form of hemophagocytic lymphohistiocytosis, is a rare life-threatening complication that may occur spontaneously as a complication of active underlying diseases such as flares in rheumatologic diseases like systemic onset juvenile idiopathic arthritis (sJIA)—a typical inflammatory disease with an overproduction of multiple cytokines such as IL-1, IL-6, IL-18, TNF-α, IL-17A, interferon γ, and others^2^ - or may be triggered by an infection, a change in drug therapy, or a toxic effect of medication^3^.

The main clinical features of MAS are the transition of intermittent fever into a continuous type, hepatosplenomegaly, lymphadenopathy, central nervous system dysfunction, purpura, mucosal haemorrhage, cytopenia, and abnormal renal function^4^. Laboratory features are decreasing white blood cells (WBC), platelet counts, fibrinogen, and erythrocyte sedimentation rate (ESR). The levels of liver enzymes, triglycerides, and ferritin are usually elevated as well^3^. The pathognomonic finding of the disease is found on bone marrow examination as numerous morphologically benign macrophages exhibiting hemophagocytic activity, which can also be observed in lymph nodes and the spleen^3^.

Diagnosis should be as prompt as possible as early initiation of treatment significantly improves the outcome. This usually begins with high-dose steroids, intravenous immunoglobulins, and immunosuppressive therapy. In recent years more advanced approaches included the use of selective cytokines inhibitors^5^.

In this report, we present a case of a 9-year-old boy with sJIA who presented with high-grade fever, vomiting, and nose bleeding. He was diagnosed with MAS and treated with antibiotics and steroids, with a good response. This case focuses light on the importance of considering MAS in patients with sJIA with flare-up signs as well as symptoms of immune dysregulation, which in turn with the adequate timely approach aids in better prognosis and outcome. The work has been reported in line with the SCARE 2023 criteria^6^.

## Case presentation

This is a case of a 9-year-old male who has been diagnosed with sJIA since the age of 6 years by a paediatric rheumatologist, with diagnostic criteria based on his previous symptoms which have been high-grade fever, arthralgia, and lymphadenopathy. He was put on low-dose oral prednisolone (5 mg) and subcutaneous methotrexate, which later became unavailable in the country for the past 2 months. Since the time of diagnosis, the patient’s condition has been stable and did not develop any severe flares that require hospitalization. He has no family history of a similar condition.

The patient attended the emergency room with fever, vomiting, and bleeding from his nose for 1 week, which was of a small amount, and high frequency, and resolved after receiving fresh frozen plasma. However, there was no bleeding from other sites. In addition, there was loss of appetite, without abdominal pain or passing of black stool.

A review of systematic complaints was unremarkable. His past surgical history included just undergoing a lymph node biopsy for diagnostic purposes.

On examination, he looked sick, and pale, with yellowish discolouration of sclera but he was vitally stable except for the fever, as shown in (Table 1). The liver was palpable 2 cm below the costal margin, the abdomen was distended with shifting dullness, and he had a scrotal swelling. Glasgow coma scale (GCS) was 15\15. No remarkable signs were found upon testing the patient’s cardiovascular, respiratory, musculoskeletal, and central nervous systems.

**Table 1:** Vital signs on arrival to ER

Variable	Value
Temperature	38
Pulse rate	100
Respiratory rate	18
Blood pressure	100\55 (50^th^ centile)

Laboratory investigation showed pancytopenia, prolonged international normalization ratio (INR), elevated liver enzymes, high serum ferritin, and lactate dehydrogenase (LDH) as shown in (Table 2). Viral screening and blood culture tests were negative. In addition, Immunologic studies were negative for anti-liver kidney microsomal antibody (ALKMA). Therefore, according to the patient’s laboratory results and clinical presentation, his case was diagnosed as MAS (Table 3).

**Table 2:** Laboratory investigation

Details:	Day 1	Day 2	Reference range
Haemoglobin (g/dl)	6.2	7.2	13–17 g/dl
MCV (fl)	58	62.5	80–100 fl
MCH (pg)	15.9	20.5	27–31 pg
MCHC (g/dl)	27.3	22	30–35 g/dl
Total white blood cells	17.5	3	4–10×10^9^/l
Neutrophiles	15.8	58	2–8×10^9^/l
Lymphocytes	7.7	35	1–4×10^9^/l
Platelets	427	176	150–400×10^9^/l
ESR	20		Less than age/2 mm/h
Albumin (g/dl)	2.6	2	35–50 g/l
Urea (mg/dl)	37	12	8–21 mg/dl
Serum creatinine (mg/dl)	0.6	0.4	0.8–1.3 mg/dl
NA+(mmol/l)	129	136	135–145 mmol/l
K+(mmol/l)	1.8	2.4	3.5–5 mmol/l
Total bilirubin (mg/dl)	5	3	2–20 µmol/l
ALT (U/l)	330	153	5–30 U/l
AST (U/l)	1040	116	5–30 U/l
Direct bilirubin	1	2.3	0–6 µmol/l
CRP (mg/l)	24	99.2	< 5 mg/l
INR	>5	1.5	0.9–1.2
PTT	>120	24.5	20–40 sec
PT	>60	19.7	11–14 sec
RBS	84		65–110 mg/dl
BFFM	Negative		
LDH (IU/l)	1034		230–460 U/l
Serum ferritin	More than 1000		12–300 ng/ml

ALT, alanine transaminase; AST, alanine aminotransferase; BFFM, blood film for malaria; CRP, C reactive protein; ESR, erythrocyte sedimentation rate; INR, international normalized ratio; K, potassium; LDH, lactate dehydrogenase; MCH, mean corpuscular haemoglobin; MCHC, mean corpuscular haemoglobin concentration; MCV, mean corpuscular volume; Na, sodium; PT, prothrombin time; PTT, partial thromboplastin time; RBS, random blood sugar.

**Table 3:** Diagnostic criteria for macrophage activation syndrome: HLH-2004—revised diagnostic guidelines for HLH10^7^

'e diagnosis of HLH can be established if one of the two criteria below is met
(1) A molecular diagnosis consistent with HLH (i.e. reported mutations found in either PRF1 or MUNC13-4), or
(2) Diagnostic criteria for HLH are fulfilled (i.e. at least five of the eight criteria listed below are present)
(a) Persistent fever
(b) Splenomegaly
(c) Cytopenia (aMecting ≥2 of 3 lineages in the peripheral blood) (i) haemoglobin < 90 g/l (in infants < 4 weeks: <100 g/l) (ii) Platelets < 100×10^9^/l (iii) neutrophils < 1.0×10^9^/l
(d) Hypertriglyceremia and/or hypoFbrinogenemia (i) fasting triglycerides ≥3.0 mmol/l (i.e. ≥265 mg/dl) (ii) fibrinogen ≤ 1.5 g/l
(e) Hemophagocytosis in bone marrow^a^ or spleen or lymph nodes, no evidence of malignancy
(f) Serum ferritin ≥500 µg/l (i.e. 500 ng/ml)
(g) Low or absent NK cell activity (according to local laboratory reference)
(h) Increased serum sIL2Rα (according to local laboratory reference)

HLH, hemophagocytic lymphocytosis; NK, natural killer.

a If hemophagocytic activity is not proven at the time of presentation, further search for hemophagocytic activity is encouraged. If the bone marrow specimen is not conclusive, material may be obtained from other organs.

Subsequently, the patient was admitted to the paediatric department and received meropenem, ciprofloxacin and vancomycin, fresh frozen plasma (FFP), liver support (vitamins K, E, and D), lactulose syrup, albumin, blood transfusion, and methylprednisolone (20 mg/kg/day). The later medicine was prescribed for three days, then an additional two doses were given upon a rheumatologist’s consultation.

After 5 days, the patient was referred to the paediatrics specialist centre due to the unavailability of ICU in the hospital of admission and further follow-up was recommended.

The Patient was admitted to the ICU in the hospital where he was transferred and then was discharged after several days on the following medicines (calvive syrup, colchicine tabs, prednisolone tabs, lactulose syrup, meropenem, vancomycin, liver support vitamin E A K D, ciclosporin) with a stable health condition and normal vital signs.

## Discussion

MAS is a potentially fatal disorder that results as a complication of multiple rheumatologic disorders including polyarticular juvenile rheumatoid arthritis, systemic lupus erythematosus, rheumatoid arthritis, sarcoidosis, dermatomyositis but is most commonly associated with sJRA and adult-onset still disease. It has also been observed following autologous bone marrow transplantation, triggered by an underlying infection, or as a consequence of therapy change, or it can also occur spontaneously. MAS is considered a subtype of secondary hemophagocytic lymphohistiocytosis (sHLH)^8–11^.

It is difficult to diagnose due to its resemblance to active sJIA inspite of the availability of diagnostic criteria. It has the same prevalence in males and females can occur across all ages and can present at any moment during the disease course including remission^11^.

The pathophysiology of this disease includes depressed natural killer (NK) cell numbers and activity, it is also associated with abnormal perforin expression, together with T lymphocyte activation leading to a cytokine storm which leads to macrophage reproduction^8,10–12^.

It has been postulated that depressed NK function would lead to the inability to clear the infection at its early stages resulting in increased lymphocytes and cytokines, which in turn will trigger macrophages. In addition, certain studies link abnormal perforin expression to the underlying disease rather than a genetic abnormality^4,8^. Another hypothesis is that a failure of apoptosis of T lymphocytes after clearance of the infection results in a cytokine storm^11^.

The disease is associated with high levels of tumour necrotic factors (TNF) and interferon (IFN-g)^12^. Nonetheless, high interlukin-18 (IL-18) levels have been found in patients with both HLH and MAS^11^. Three studies concluded that high IL-18 levels were found in soJIA patients with a history of MAS whether they were in the active or inactive stages of the disease^9^.

The most sensitive presenting symptom is high fever; however, it is not specific. Other features include hepatosplenomegaly, coagulopathy, encephalopathy, and rash^8,10–12^. laboratory findings include hyperferritinemia, hypertriglyceridemia, high lactate dehydrogenase, hypofibrinogenemia, cytopenia, low ESR, and deranged liver enzymes^8,10–13^.

Unlike the paediatric population, hyperferritinemia is not a specific marker in the adult population^12^. The characteristic feature of this disease is finding differentiated macrophages or histiocytes actively phagocytosing hematopoietic elements in bone marrow aspirate, nonetheless, this is not crucial for diagnosis^8,10,13^.

MAS is managed mainly by steroids, other options include NSAIDs, cyclosporin A, etoposide, intravenous immunoglobulin, and biological agents such as anakinra^11,13–15^.

The disease can progress to multi-organ failure and death. Markers of poor prognosis include the presence of other comorbidities, severe neutropenia, coagulopathy, encephalopathy, and poor response to medications^10,11^.

Our patient fulfils the criteria required for the MAS diagnosis, with two clinical criteria, which are persistent high-grade fever and mucosal bleeding. It is worth mentioning that he has hepatomegaly that does not meet the clinical criteria. Nonetheless, he fulfills four laboratory criteria which are cytopenia affecting two cell lineages, high LDH, high alanine aminotransferase, and high ferritin (Table 4).

**Table 4:** Diagnostic criteria for macrophage activation syndrome: Parodi’s preliminary diagnostic guidelines for MAS as a complication of juvenile SLE^16^

'e diagnosis of MAS requires the simultaneous presence of at least 1 clinical criterion and at least 2 laboratory criteria
Clinical criteria
(1) Fever (>38°C)
(2) Hepatomegaly (≥3 cm below the costal arch)
(3) Splenomegaly (≥3 cm below the costal arch)
(4) Haemorrhagic manifestations (purpura, easy bruising, or mucosal bleeding)
(5) Central nervous system dysfunction (irritability, disorientation, lethargy, headache, seizures, or coma)
Laboratory criteria
(1) Cytopenia affecting 2 or more cell lineages (white blood cell count ≤4.0×10^9^/l, haemoglobin ≤90 g/l, or platelet count ≤150×10^9^/l)
(2) Increased aspartate aminotransferase (>40 U/l)
(3) Increased lactate dehydrogenase (>567 U/l)
(4) HypoFbrinogenemia (fibrinogen ≤1.5 g/l)
(5) Hypertriglyceridemia (triglycerides >178 mg/dl)
(6) Hyperferritinemia (ferritin >500 µg/l)
Histopathologic criterion
Evidence of macrophage hemophagocytosis in the bone marrow aspirate^a^

MAS, macrophage activation syndrome.

a Bone marrow aspiration for evidence of macrophage hemophagocytosis may be required only in doubtful cases.

Previous similar studies were reviewed from multiple journals and scientific search engines, (e.g. PubMed), with “sJIA and MAS” used as keywords for searching similar case reports and reviews.

A similar case has been reported in a previous case of a 3-year-old male patient born to consanguineous parents, with a history of Griscelli syndrome type 2^5^. Upon clinical examination hepatosplenomegaly was evident. Moreover, laboratory investigations showed pancytopenia, hyperferritinemia, and hypofibrinogenemia^5^. A similar management plan was carried out, which was giving parenteral steroids which unfortunately was of no benefit, hence the regimen was changed to ectopside with cyclophosphamide resulting in satisfactory improvement evident by declining ferritin levels and macrophages with hemophagocytic activity^5^. The suspected infectious process was managed by antibiotics and was discharged on prednisolone and cyclophosphamide^5^. An additional management step was made which is placing the patient on the bone marrow transplantation list, particularly attributed to his pancytopenia, however, this procedure is not available in Sudan.

Another reported case is a case of a 17-month-old female patient born to consanguineous parents who presented with a combination of Visceral leishmaniasis and MAS^17^. Diagnosing MAS in this patient was challenging because of the similarity of these two conditions. Clinically, this patient had a high-grade fever, petechiae, and hepatosplenomegaly. Blood counts revealed pancytopenia, as well as hypertriglyceridemia, hyperferritinemia, hypofibrinogenemia, and high lactate dehydrogenase. Bone marrow examination revealed features of hemophagocytosis and the diagnosis of visceral leishmaniasis with MAS was made. She was managed with liposomal amphotericin B coupled with corticosteroid therapy resulting in a favourable outcome^17^.

An additional study reported MAS in a healthy 39-year-old lady with no previous medical history unlike our case and the previously reported case^18^. This lady presented with hemodynamic instability. She had similar laboratory findings of hypertriglyceridemia, pancytopenia, hyperferritinemia, high lactate dehydrogenase, and high alanine aminotransferase. The immunological and infectious screening was negative^18^. Bone marrow biopsy revealed normal cellularity, activated monocytes, and initial hemophagocytosis. The patient was diagnosed with MAS and was given immunoglobulins and cyclosporine along with supportive management and antibiotics; however, the patient progressed to develop multi-organ failure and death^18^.

Similarities and variabilities between all these cases may aid in generating a potential for guidelines on diagnosis and management as well as focusing the light on the degree of variabilities between the cases and its impact on the prognosis and outcome of patients.

Financial limitations, as well as the unavailability of certain tests and equipments, were the hallmark limitations in the comprehensive approach towards this case. However, it is recommended that further research and deliberate investigations be carried out for more authentic and evidence-based data regarding MAS.

## Conclusion

MAS is a real life-threatening complication for patients with sJIA. Early diagnosis and timely treatment can both offer a favourable result against such a syndrome. In addition, further research and reviews should be conducted to generate evidence-based data regarding the approach to diagnosis and management of MAS.

## Ethical approval

Ethical approval was not needed as this is a case report.

## Consent for publication

Written informed consent was obtained from the patient’s parents for the publication of this case report and accompanying images. A copy of the written consent is available for review by the Editor-in-Chief of this journal on request.

## Sources of funding

The study was funded by the authors themselves.

## Author contribution

M.A.Y.E., R.M.H.I., B.M.S.M.E., N.S.B.S. wrote the first draft, visualized, validated, conceptualized, did the investigations, and administered the project, while R.A.O.M., M.M.F.E., G.E.M. and L.H. wrote the first draft, visualized, validated, conceptualized, and critically reviewed, and K.A.H.M.A. wrote the first draft, validated, visualized, conceptualized, and supervised the study. All authors participated in the examination, investigation, management and writing of this case.

## Conflicts of interest disclosure

No competing of Interest is to be declared.

## Research registration unique identifying number (UIN)

1. Name of the registry: N/A.
2. Unique Identifying number or registration ID: N/A.
3. Hyperlink to your specific registration (must be publicly accessible and will be checked):

## Guarantor

Khabab Abbasher Hussien Mohamed Ahmed.

## Data availability statement

The dataset used and/or analyzed during the study is available from the corresponding author upon reasonable request.

## Provenance and peer review

Not commissioned, externally peer-reviewed.
